# The significance of LRPPRC overexpression in gastric cancer

**DOI:** 10.1007/s12032-013-0818-y

**Published:** 2013-12-28

**Authors:** Xiaosa Li, Lifen Lv, Jianyong Zheng, Jinfeng Zhou, Bing Liu, Hui Chen, Cong Liang, Rui Wang, Linna Su, Xiaohua Li, Daiming Fan

**Affiliations:** State Key Laboratory of Cancer Biology, Xijing Hospital of Digestive Diseases, Fourth Military Medical University, 127# Changle West Road, Xi’an, 710032 China

**Keywords:** LRPPRC, LRP130, Gastric cancer, Expression, Prognosis

## Abstract

LRPPRC is a multifunctional protein involved in mitochondrial gene expression and function, cell cycle progression, and tumorigenesis. We analyzed LRPPRC gene expression in 253 paired cases of gastric cancer and noncancerous regions and six gastric cancer cell lines to demonstrate the importance of LRPPRC expression for the prediction of prognosis of gastric cancer. Our results showed that LRPPRC expression in gastric cancer tissues is significantly higher than that in paired control tissue (*P* < 0.001). Patients with higher LRPPRC expression showed a poorer overall survival rate than those with lower LRPPRC expression (*P* < 0.001). Multivariate analysis demonstrated that lymph node metastasis (N), distant metastasis (M), TNM stage, and LRPPRC expression were independent prognostic factors for gastric cancer (*P* = 0.004, 0.002, 0.017, 0.004 respectively).Moreover, Western blotting showed that LRPPRC expression was increased in SGC7901, BGC823, MKN45, and XGC9811cells. The in vitro proliferation assay showed that LRPPRC expression is inversely associated with gastric cancer cells growth. Our results indicated that LRPPRC could be used as a predictive marker for patient prognosis of gastric cancer and may be a novel therapeutic target for gastric cancer in future.

## Introduction

Gastric cancer is a disease with one of the poorest prognoses, being the second cause of tumor-related mortality in the world. Five-year overall survival is 25 % or less, especially in USA, Europe, and China [1, 2]. Every year, 1 million new cases of gastric cancer are diagnosed and 700,000 die of this disease worldwide [3, 4].Most patients with gastric cancer are diagnosed with advanced gastric cancer, and overall survival rate remains poor. To provide new insights into the pathology of the disease and to permit earlier diagnosis, there is a need for new prognostic tumor markers that are more sensitive than those currently available, such as CEA and CA19-9 [5].

In 2000, Small and Peeters [6] described a set of proteins with 35 amino acid repeat sequences that were dubbed ‘pentatricopeptide repeat cassette proteins’. Members of the pentatricopeptide repeat (PPR) protein family play important roles in mitochondrial RNA metabolism in metazoans, plants, and yeast [7]. LRPPRC protein (also known as LRP130) [8, 9], a member of PPR protein family, regulates the stability and handling of mature mitochondrial mRNA and participates in the formation of the transcriptional activator PGC-1 involved in liver glucose homeostasis, energy metabolism, and nuclear receptor activation [10].

Previous reports have showed that LRPPRC was highly expressed in most cancers, such as hepatoma cancer, lung adenocarcinoma, esophageal squamous cell carcinoma, colon cancer and lymphoma, and significantly associated tumorigenesis and invasion [11]. However, the LRPPRC expression in gastric cancer and its correlation with gastric cancer clinicopathological characteristics is still unclear.

In this study, we first examined LRPPRC expression in 253 paired cases of gastric cancer and paired noncancerous regions and six gastric cancer cell lines to investigate the relevance of LRPPRC expression and its relation to clinicopathological characteristics. Besides, an in vitro study was performed to observe the LRPPRC effect on gastric cancer cell proliferation. In conclusion, our results suggested that LRPPRC is a novel independent marker for the prognosis with functional relevance in gastric cancer.

## Materials and methods

### Clinical tissue samples

Our study included 253 patients (153 males, 100 females; mean age 65.5 years; range 34–83), who underwent surgery at Xijing Hospital, Fourth Military Medical University (Xi’an, China), were recruited between May 2003 and August 2005 after obtaining their written informed consent. Cancer tissues, along with normal tissues that were at least 5 cm away from the cancer, were obtained from the patients. The median follow-up period for survivors was 47 months (range 0–128 months) by telephone and mail. The study items included age, gender, location of the tumor, tumor stage, depth of invasion, lymph node metastasis, distant metastasis, and tumor-node-metastasis (TNM) stage. Patient characteristics are summarized in Table 1. All of the patients were staged using the 7th edition of the International Union Against Cancer TNM staging system. Of the 253 patients, 66(26 %) had T1-stage, 60(24 %) had T2-stage, 84(33 %) had T3-stage, and 43(17 %) had T4-stage gastric cancer. Tissues were fixed in 10 % formaldehyde, embedded in paraffin, cut into 4-μm sections, and mounted on slides.

**Table 1:** Correlation between clinicopathological characteristics and LRPPRC expression

Total	*n*	LRPPRC (±)	LRPPRC (++)	LRPPRC (+++)	*P*
Age					0.617^a^
≤65.5	131	41	48	42	
>65.5	122	40	35	47	
Gender					0.357^a^
Male	153	51	52	50	
Female	100	30	31	39	
Location					0.905^b^
Gastric antrum	105	39	26	40	
Lessercurvature	75	23	27	25	
Gastric cardia	73	19	30	24	
T					<0.001^b^
T1	66	42	17	7	
T2	60	23	29	8	
T3	84	9	27	48	
T4	43	7	10	26	
N					<0.001^b^
N0	58	32	18	8	
N1	63	25	27	11	
N2	97	19	29	49	
N3	35	5	9	21	
M					0.002^a^
M0	219	76	74	69	
M1	34	5	9	20	
TNM					0.880^b^
1	33	11	13	9	
2	92	26	34	32	
3	101	35	29	37	
4	27	9	7	11	
Tumor grade					0.748^b^
Well	34	11	14	9	
Moderately	138	43	44	51	
Poorly	81	27	25	29	

^a^ *P* value for expression levels compared by Mann–Whitney test

^b^ *P* value for expression levels compared by Kruskal–Wallis test

### Immunohistochemical staining

The cancer and noncancerous tissues from 253 patients were embedded in paraffin and cut into sections for immunohistochemical analysis. Slides were baked at 60 °C for 2 h, followed by deparaffinization with xylene, and rehydrated, after being washed three times in PBS (phosphate-buffered saline), then using a pressure cooker with 10 nM citrate buffer (PH 6.0) for 5 min. After rinsing with PBS. They were then treated with 3 % hydrogen peroxide for 12 min in methanol to quench endogenous peroxidase activity, followed by incubation with 1 % bovine serum albumin to block nonspecific binding for 1 h. The antigen–antibody reaction was carried out overnight at 4 °C with the anti-LRPPRC antibody diluted 1:500(Santa Cruz Biotechnology Inc., Santa Cruz, CA). Rinsed for three times in PBS and incubated with a horseradish-peroxidase-conjugated anti-IgG antibody (1:3,000; Santa Cruz) for 1 h. Finally, the sections were developed with 3,3′-diaminobenzidine solution for 2 min, washed briefly in running water, counterstained with hematoxylin, dehydrated through a graded series of alcohol to xylene and were then mounted with Permount onto coverslips. Images were obtained under a light microscope (Olympus BX51;Olympus, Japan) equipped with a DP70 digital camera. As negative controls, tissue sections were processed under the same experimental conditions described above, except that they were incubated overnight at 4 °C in blocking solution without the anti-LRPPRC antibody.

### Immunohistochemical analysis

Staining of LRPPRC was detected mainly in the cytoplasm of tumor cells. The degree of immunostaining was reviewed and scored independently by two pathologists who did not know the clinical features or survival status of the patients then viewed the stained tissue slides separately. An average value of two independent scores was presented in the present study [12–14]. Expression of LRPPRC was evaluated according to the ratio of positive cells per specimen and staining intensity. The ratio of positive cells per specimen was evaluated quantitatively and scored as follows: 0 = staining of ≤1 %;1 = staining of 2–25 %; 2 = staining of 26–50 %; 3 = staining of 51–75 %; and 4 = staining of >75 % of the cells examined. Intensity was graded as follows: 0 = no signal; 1 = weak; 2 = moderate; and 3 = strong. A total score of 0–12 was finally calculated and graded as negative (−; score: 0–1), weak (+; score: 2–4), moderate (++; score: 5–8), and strong (+++; score: 9–12) [14, 15].

### Cell culture, plasmid construction, and cell transfection

Gastric cancer cell lines (KATOIII, SGC7901, BGC823, MKN45, MKN28, and XGC9811) were maintained in Dulbecco’s modified Eagle’s medium (Gibco RL, Grand Island, NY) supplemented with 10 % fetal bovine serum, 100 U/ml penicillin, and 0.1 mg/ml streptomycin. And incubated at 37 °C, 5 %CO_2_. For the small interference RNA (siRNA)-knockdown experiment, double-stranded RNA duplexes that targeted the human LRPPRC gene (5′-CACCGGAGGAGCATTTGAGACAATATTCAAGAGATATTGTCTCAAATGCTCCTCCTTTTTTG-3′/5′-GATCCAAAAAAGGAGGAGCATTTGAGACAATATCTCTTGAATATTGTCTCAAATGCTCCTCC-3′) were synthesized, negative control (NC) siRNA was also synthesized. Gastric cancer cell lines were transfected with siRNA at concentration of 20 lmol/L with lipofectamine (RNAiMAX, Invitrogen), incubated in glucose-free Opti-MEM (Invitrogen) for the time indicated, and analyzed by the proliferation assay. All siRNA duplexes were used together as a triple transfection. siRNA knockdowns were performed in four Gastric cancer cell lines to evaluate proliferation value under LRPPRC suppression. The values are presented as mean ± standard deviation (SD) from independent experiments conducted in triplicate.

### Western blot

Cells were washed twice with cold PBS and lysed on ice in RIPA buffer with protease inhibitors and quantified by BCA method. 50 mg Protein lysates were resolved on 8 % SDS polyacrylamide gel, electrotransferred to polyvinylidene fluoride membranes (Millipore, Bedford, MA) and blocked in 5 % nonfat dry milk in Tris-buffered saline (pH = 7.5). Membranes were immunoblotted overnight at 4 °C with anti-LRPPRC polyclonal antibodies as IHC described above, respectively, then followed by their respective secondary antibodies. Signals were detected by enhanced chemiluminescence (Pierce, Rockford, IL). For Immunofluorescence, the binding of primary antibody was visualized by anti-rabbit IgG antibody, and the slides were then examined by a confocal laser scanning microscope.

### Proliferation assays

In gastric cancer cell lines transfected with siRNA, 1 × 10^5^ cells were seeded in 12-well dishes and cultured for 96 h to determine proliferation. Viable cells were counted every day by reading the absorbance at 490 nm using a 96-plate reader BP800 (Dynex Technologies, Chantilly, VA, USA). Each experiment was performed in triplicate.

### Statistical analysis

All statistical analyses were performed using the SPSS⑨(QUANER) version 16.0 software package (SPSS Inc. Chicago, IL, USA). A paired samples *t* test was used to analyse the differences between the gastric cancer samples and the paired adjacent noncancerous tissue samples. Associations between LRPPRC expression and clinicopathological characteristics were analyzed by the Mann–Whitney test and the Kruskal–Wallis test. Survival curves were estimated using the Kaplan-Meyer method, and the log rank test was used to calculate differences between the curves. Prognostic factors were examined by univariate and multivariate analyses (Cox proportional hazards model). A probability level of 0.05 was chosen for statistical significance.

## Results

### LRPPRC expression in clinical tissue specimens

**Fig. 1:**
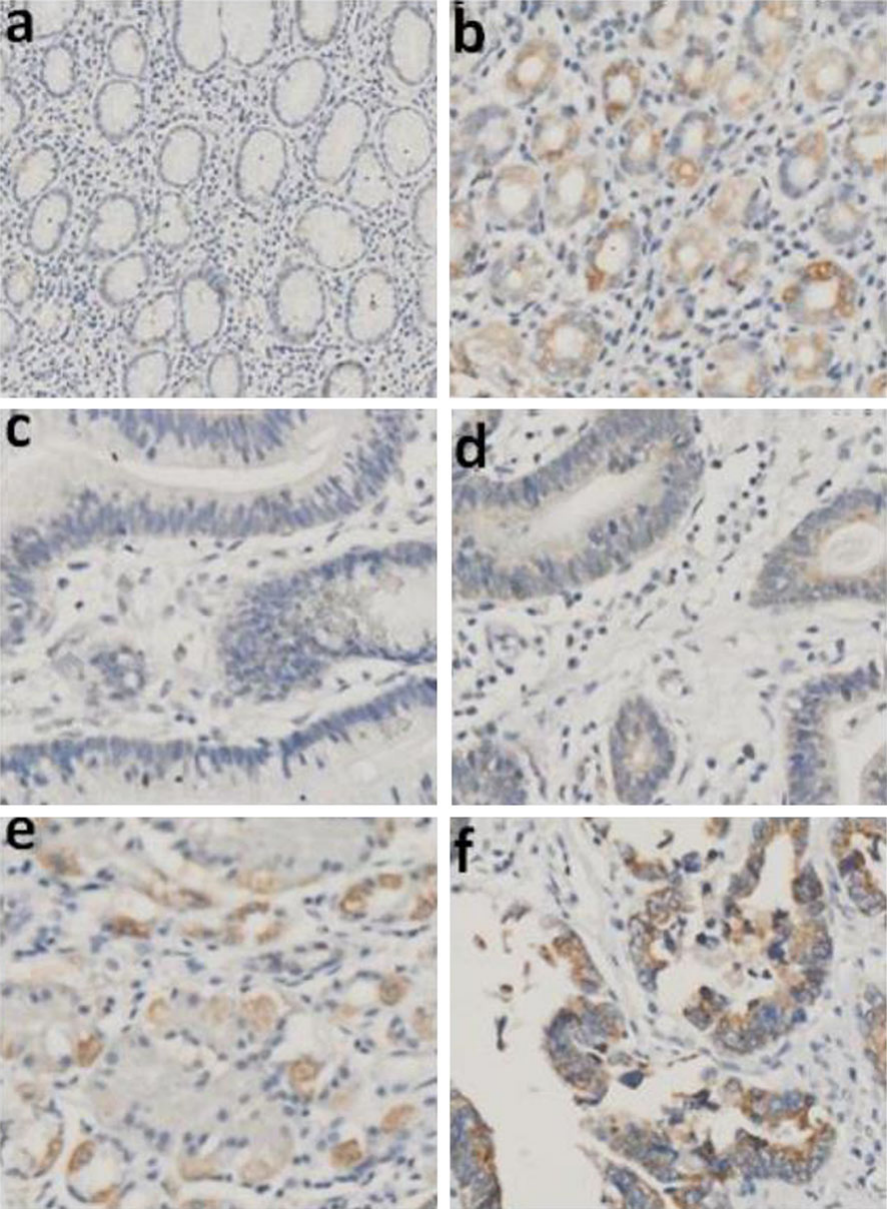
Immunohistochemical staining of LRPPRC in GC tissues (original magnification, ×200).**a** Negative staining (−) of LRPPRC in normal mucosa. **b** Moderate staining (++) of LRPPRC in normal mucosa. **c** Negative staining (−)of LRPPRC in GC tissue. **d** Weak staining (+) of LRPPRC in GC tissue. **e** Moderate staining (++) of LRPPRC in GC tissue. **f** Strong staining (+++) of LRPPRC in GC tissue

LRPPRC expression was investigated by immunohistochemistry in 253 gastric cancer tissues and paired noncancerous tissues. We found that positive LRPPRC expression in gastric cancer tissues (219/253, 86.6 %) was significantly higher than that in paired noncancerous tissues (132/253, 52.2 %). The difference in LRPPRC staining between gastric cancer tissues and paired noncancerous tissues was statistically significant (*P* < 0.001) (Fig. 1). In details, positive immunoreactivity was observed in 86.6 % gastric cancer tissues, with 47 (21.4 %) displaying weak (+) positive expression, 83(38 %) moderate (++) positive expression, and 89(40.6 %) strong (+++) positive expression. In contrast, in the paired noncancerous tissues, negative expression of LRPPRC was detected in 47.8 % (121/253) of the specimens.

### LRPPRC expression and clinicopathological characteristics

According to LRPPRC expression status, we divided the gastric cancer samples into three groups for the clinicopathological evaluation. The correlation between LRPPRC and clinicopathological features in gastric cancer was further evaluated. The number of cases that were based on histological grade was 34, 138, and 81 in the well, moderate, and poor, respectively. The results showed that increased expression of LRPPRC was significantly correlated with the depth of tumor infiltration (T stage, *P* < 0.001, N stage *P* < 0.001 and M stage *P* = 0.002), whereas not with age, gender, tumor locus, tumor grade and TNM stage.

### Relationship between LRPPRC expression and prognosis

**Fig. 2:**
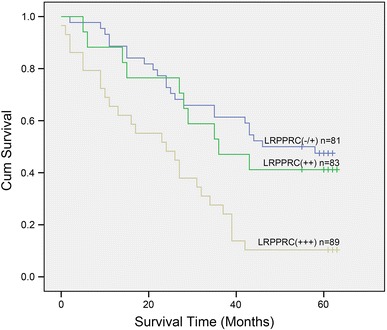
Kaplan–Meier postoperative survival curve for patterns with gastric cancer and LRPPRC expression

The overall survival analysis using the Kaplan-Meyer method revealed that the prognosis of gastric cancer patients whose tumors with higher or moderate LRPPRC expression showed significantly shorter survival than those with no or weak LRPPRC expression (*P* < 0.001; Fig. 2). Table 2 provides the univariate and multivariate analyses of factors related to patient prognosis. Univariate analysis shows that the following factors were significantly related to postoperative survival: depth (*P* = 0.004), lymph node metastasis (*P* = 0.007), distant metastasis (*P* < 0.001), TNM stage (*P* = 0.004), and LRPPRC expression (*P* < 0.001). Furthermore, a Multivariate analysis indicated that lymph node metastasis (N *P* = 0.004), distant metastasis (M *P* = 0.002), TNM stage (*P* = 0.017), and LRPPRC expression (*P* = 0.004) were independent prognostic factors of overall survival for the patients with gastric cancer.

**Table 2:** Univariate and multivariate analyses of overall survival of gastric cancer patients

Variables	Univariate analyses		*P*	Multivariate analyses		*P*
	HR	(95 % CI)		HR	(95 % CI)	
Age			0.055			
≤65.5	1.000					
>65.5	1.658	0.990–2.776				
Gender			0.906			
Male	1.000					
Female	1.035	0.583–1.838				
Location			0.909			
Gastric antrum	1.000					
Lessercurvature	1.067	0.551–2.066				
Gastric cardia	1.147	0.614–2.145				
T			0.004*			0.094
T1	1.000			1.000		
T2	1.056	0.096–11.643		1.100	0.081–14.873	
T3	3.913	0.537–28.492		1.603	0.201–12.770	
T4	8.575	1.131–64.990		3.822	0.460–31.752	
N			0.007*			0.004*
N0	1.000			1.000		
N1	1.546	0.613–3.899		1.300	0.476–3.550	
N2	2.104	0.943–4.696		1.343	0.393–4.590	
N3	3.601	1.653–7.841		4.635	1.321–16.265	
M			<0.001*			0.002*
M0	1.000			1.000		
M1	7.098	3.104–16.230		4.792	1.739–13.204	
TNM			0.004*			0.017*
1	1.000			1.000		
2	1.957	0.562–6.812		8.991	2.208–36.611	
3	3.120	0.745–13.063		9.819	1.878–51.347	
4	4.892	1.500–15.946		10.847	2.057–57.213	
Tumor grade			0.190			
Well	1.000					
Moderately	1.089	0.442–2.688				
Poorly	1.718	0.716–4.124				
LRPPRC			<0.001*			0.004*
±	1.000			1.000		
++	1.230	0.585–2.585		1.060	0.475–2.365	
+++	3.012	1.698–5.341		2.883	1.472–5.647	

*HR* hazard ratio, *CI* confidence interval, a Numbers of cases in each group; * statistically significant (*P* < 0.05)

### In vitro assessment of LRPPRC expression knockdown

**Fig. 3:**
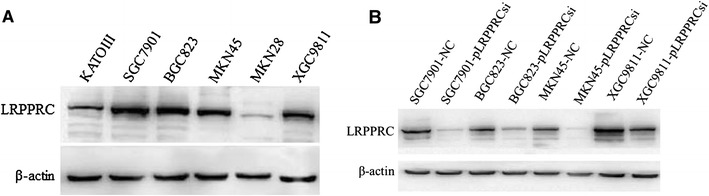
**a** Expression of LRPPRC in the gastric cancer cell lines. β-actin was used as an internal control. The level of LRPPRC protein expression was significantly higher in the gastric cancer cell line SGC7901, BGC823, MKN45, and XGC9811 than in other cell lines. **b** Expression of LRPPRC siRNA in transfected cells detected by Western blot analysis

**Fig. 4:**
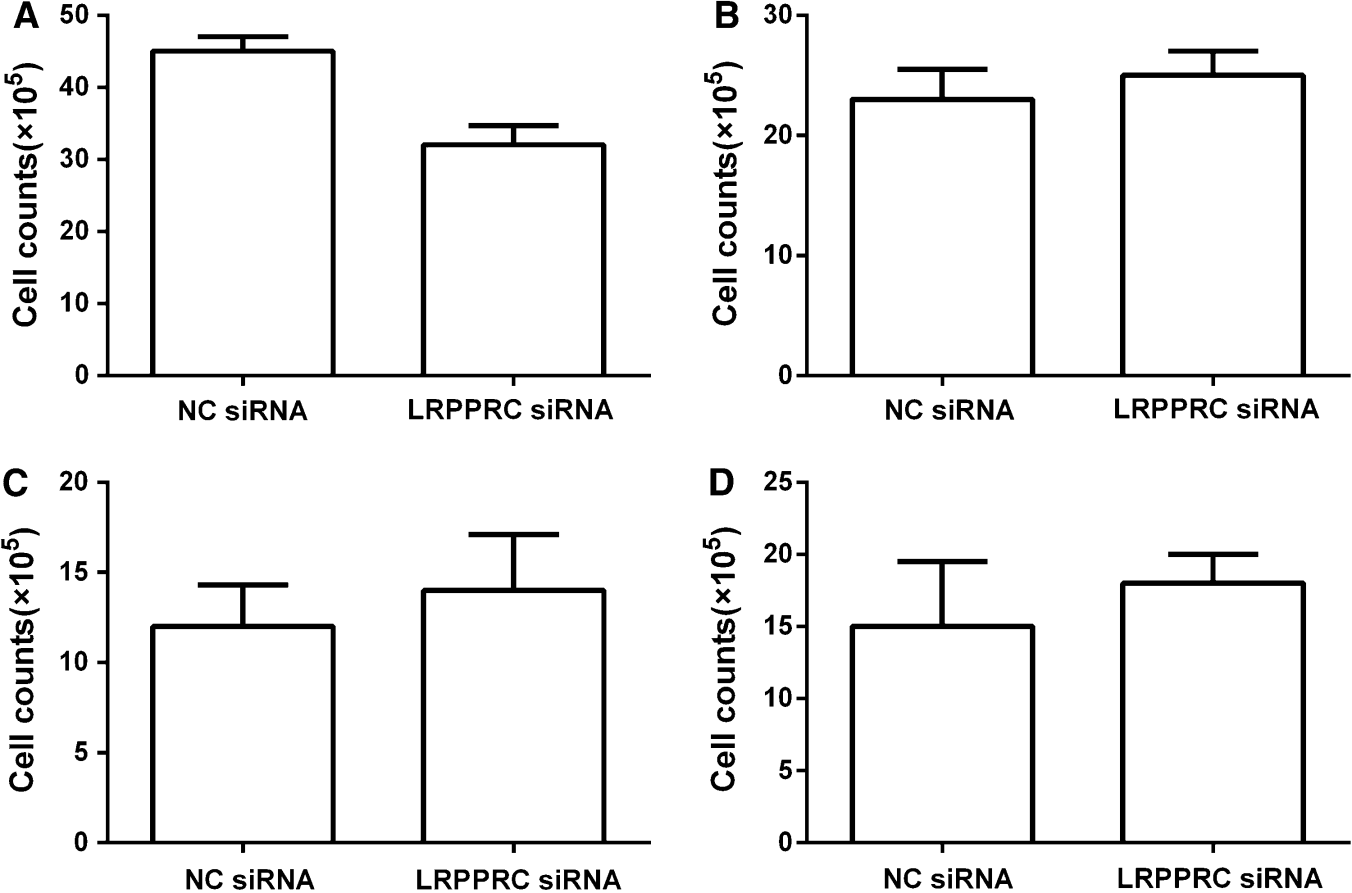
Proliferation assay and siRNA inhibition in 4 gastric cancer cell lines. The proliferation assay showed a difference in growth of gastric cancer cell line SGC7901. There were significant differences between NC and LRPPRC siRNA. In the other 3 cell lines, there was no significant difference between NC and LRPPRC siRNA (**a** SGC7901; **b** BGC823; **c** MKN45; **d** XGC9811). Values are mean ± SD for three independent experiments. *NC* negative control

Because LRPPRC expression was higher in gastric cancer tissues than that in paired noncancer tissues, six gastric cancer cell lines were chosen for the proliferation study. We first examined the expression of LRPPRC in six gastric cancer cell lines by Western blot. Our results showed that LRPPRC protein level was higher expressed in gastric cancer cell line SGC7901, BGC823, MKN45, and XGC9811 (Fig. 3a) as compared with the other gastric cancer cell lines. After cell transfection, the expression of LRPPRC in transfected cells was determined by Western blotting. It was found that LRPPRC expression was significantly reduced in SGC7901,BGC823,MKN45, and XGC9811 (Fig. 3b). In proliferation assay, there were differences in cell numbers of SGC7901 between NC and LRPPRC siRNA (*P* < 0.05; Fig. 4). There was no statistically significant difference in the number between the NC and LRPPRC siRNA in the other cell lines.

## Discussion

Gastric cancer is usually a disease of the aged, with the mean patient age ranging between 50 and 70 years. It is thought that gastric cancer results from a combination of environmental factors and an accumulation of generalized and specific genetic alterations [16]. The treatment for gastric cancer includes a combination of surgery, chemotherapy, and radiation therapy. There have been some studies on the prognostic impact of tumor markers in gastric cancer, but the previous studies have not evaluated the relevance in LRPPRC expression and tumor prognosis. The assessment of biological prognostic factors is of clinical importance, especially for a disease with poor outcome such as gastric cancer.

The primary aim of this study was to determine LRPPRC expression and its correlation with clinicopathological, characteristics, and prognosis of patients with gastric cancer. To investigate the potential oncogenic role of LRPPRC in gastric cancer, we first examined the expression level of LRPPRC in a series of paired gastric cancer tissues with the adjacent nonneoplastic tissues. The expression of LRPPRC was verified by immunohistochemistry in gastric cancer and corresponding normal tissues. Results showed that the LRPPRC expression levels were significantly increased in tumor tissue samples, compared with that in the adjacent nontumor tissue samples, as illustrated in Fig. 1. The similar result was obtained in other studies showing that LRPPRC protein was indeed relatively upregulated in gastric cancer and others carcinoma tissues. ^16^In addition, according to analysis of the correlation between expression level of LRPPRC and patients’ characteristics, one of the interesting findings was that expression level of LRPPRC was significantly associated with the depth of tumor invasion, lymph nodes metastasis (N stage), and distant metastasis (M stage) (Table 1).Tian et al. [11] reported that the lung adenocarcinoma cell line A549, treated with LRPPRC, had high invasive ability. The present in vitro study showed that LRPPRC expression is associated with tumor growth, and the inhibition of LRPPRC may lead to a reduction in gastric cancer proliferation. These results suggest that LRPPRC may play an important role in the tumorigenesis of gastric cancer (Fig. 4).

One of the most important findings in this study was that high expression of LRPPRC in gastric cancer patients was significantly associated with poor prognosis and low overall survival, as shown in Fig. 2, indicating that high LRPPRC protein level is a marker of poor prognosis for patients with gastric cancer. Multivariate analyses in Table 2 further revealed that only lymph node metastases and distant metastases, TNM stage, and expression of LRPPRC were independent prognostic factors in patients with gastric cancer.

In summary, our results show that high LRPPRC protein expression is correlated with the depth of tumor infiltration and is an unfavorable independent prognostic factor for surgically resected gastric cancer. The metastasis mechanism of LRPPRC in gastric cancer may involve a multitude of epigenetic pathways and needs to be addressed in the future. These findings suggest that LRPPRC can serve as a predictive marker of patient outcome in gastric cancer. Since the number of patients in this study was small, further study of a larger patient population is necessary to confirm its clinical significance in gastric cancer.
